# The Mammalian “Obesogen” Tributyltin Targets Hepatic Triglyceride Accumulation and the Transcriptional Regulation of Lipid Metabolism in the Liver and Brain of Zebrafish

**DOI:** 10.1371/journal.pone.0143911

**Published:** 2015-12-03

**Authors:** Angeliki Lyssimachou, Joana G. Santos, Ana André, Joana Soares, Daniela Lima, Laura Guimarães, C. Marisa R. Almeida, Catarina Teixeira, L. Filipe C. Castro, Miguel M. Santos

**Affiliations:** 1 CIMAR/CIIMAR-AL, Interdisciplinary Centre of Marine and Environmental Research, Rua dos Bragas 289, 4050–123, Porto, Portugal; 2 FCUP–Department of Biology, Faculty of Sciences, University of Porto, Porto, Portugal; Karlsruhe Institute of Technology, GERMANY

## Abstract

Recent findings indicate that different Endocrine Disrupting Chemicals (EDCs) interfere with lipid metabolic pathways in mammals and promote fat accumulation, a previously unknown site of action for these compounds. The antifoulant and environmental pollutant tributyltin (TBT), which causes imposex in gastropod snails, induces an “*obesogenic*” phenotype in mammals, through the activation of the nuclear receptors retinoid X receptor (RXR) and peroxisome proliferator-activated receptor gamma (PPARγ). In teleosts, the effects of TBT on the lipid metabolism are poorly understood, particularly following exposure to low, environmental concentrations. In this context, the present work shows that exposure of zebrafish to 10 and 50 ng/L of TBT (as Sn) from pre-hatch to 9 months of age alters the body weight, condition factor, hepatosomatic index and hepatic triglycerides in a gender and dose related manner. Furthermore, TBT modulated the transcription of key lipid regulating factors and enzymes involved in adipogenesis, lipogenesis, glucocorticoid metabolism, growth and development in the brain and liver of exposed fish, revealing sexual dimorphic effects in the latter. Overall, the present study shows that the model mammalian *obesogen* TBT interferes with triglyceride accumulation and the transcriptional regulation of lipid metabolism in zebrafish and indentifies the brain lipogenic transcription profile of fish as a new target of this compound.

## Introduction

*“Obesogens”* are a class of Endocrine Disrupting Chemicals (EDCs) present in the environment, named after their potential to induce obesity in mammals [1, 2]. Some of these chemicals, such as organotins, bisphenol A and phthalates, with very diverse chemical structures, were initially characterized as “androgenic” or “estrogenic” due to their ability to mimic or alter the endogenous role of androgens or estrogens, leading to reproductive dysfunction [3–5]. However, recent evidence suggest that these compounds also interfere with nuclear receptors that play a key role in adipogenesis, sometimes at concentrations much lower than the ones reported to alter the hormone synthesis and metabolism, resulting in fat accumulation [1, 2, 6].

Tributyltin (TBT) is an organotin compound that was used globally as a biocide in ships’ antifouling paints. Continuous evidence on its high toxicity towards a wide range of aquatic organisms resulted in its ban and total prohibition of use in antifouling paints in 2008 [4, 7, 8]. Organotins are also used in wood preservation, as antifungal agents in breweries, textiles, wood pulp, paper mill and industrial water systems, as well as a heat and light stabilizers for PVC material [7]. Due to their low solubility, high octanol-water partition coefficient and persistence in sediments, their release into the aquatic environment has resulted in worldwide aquatic contamination [7, 9]. Despite its ban in antifouling paints, TBT is still detected in water samples near harbours at concentrations up to 200–400 ng/L [9, 10] and in sediments up to 1–10 μg/ L [10, 11].

Several studies have identified endocrine system abnormalities related to TBT exposure in a wide range of organisms including molluscs [4, 12, 13], fish [14–16] and mammals [17–19]. One of the most studied cases in endocrine disruption research is the imposition of male secondary sexual characteristics in female gastropods (imposex), an irreversible condition that develops following exposure to TBT at concentrations as low as 1 ng/L (as Sn) [20]. TBT binds to and activates the molluscan retinoid X receptor (RXR) [21, 22] and the human RXR ligand 9-*cis* retinoic acid (9-cis RA) induces imposex to female gastropods at nanomolar concentrations [22]. These data strongly suggest that RXR modulation is the primary mechanism of TBT imposex induction in gastropods [23].

In mammals, TBT is a nanomolar affinity ligand of RXRα and peroxisome proliferator-activated receptor gamma (PPARγ) [17, 24, 25]. PPARγ, which forms an obligatory permissive heterodimer with RXR, is considered a “*master*” regulator of adipogenesis since its activation enhances cellular processes involved in adipocyte maturation by inducing the expression of genes mediating fatty acid uptake, metabolism and storage [26]. Indeed, *in vitro* studies show that exposure of rodent or human undifferentiated adipocyte cell lines (3T3-L1) and bone marrow multipotent mesenchymal stromal cells (MSCs) to TBT or RXRα- and PPARγ-specific ligands results in adipocyte differentiation and triglyceride accumulation with a parallel induction of the expression of adipogenic marker genes [17, 24, 27, 28]. TBT also stimulates mammalian adipogenesis *in vivo* when exposure occurs during the pre-natal and early post-natal stages. Exposed mice show increased adipose mass and liver lipid accumulation [17, 18], accompanied by alterations in the expression of key factors and enzymes of the lipid metabolism in liver and adipose tissue [19]. The common pattern of TBT on PPARγ:RXR heterodimer target genes, both *in vitro* and *in vivo*, strongly indicate that TBT perturbs lipid metabolism favouring obesity through the activation of nuclear receptors [17, 24, 29].

Fish are continually exposed to endocrine disruptors in their natural environment and are sensitive to TBT exposure. TBT effects upon fish include altered sex ratio, suppressed fertility, inhibition of neural aromatase gene expression and alterations in the signalling of hepatic key regulatory steroidogenic enzymes [14, 15, 30, 31]. Despite the evidence on the adverse action of TBT in fish, there are limited data on the effects of this compound on fat accumulation and on the transcription network that regulates lipid metabolism. Lipid absorption, transport, storage and metabolism in fish are achieved through biological and molecular processes that resemble that of mammalian species [32, 33]. Furthermore, fish may develop human-like metabolic diseases such as the obesity-related non-alcoholic fatty liver disease (NAFLD) and hepatic steatosis [34–36]. Additionally, the mammalian targets of TBT, RXRα and PPARγ, have clear orthologues in fish, with their expression detected since early development [37, 38]. Thus, TBT and other chemicals, may act on the transcriptional regulation of lipogenesis in fish and induce either “*obesogenic*” or overall lipid deregulation responses, posing a yet unidentified threat to fish populations.

**Fig 1:**
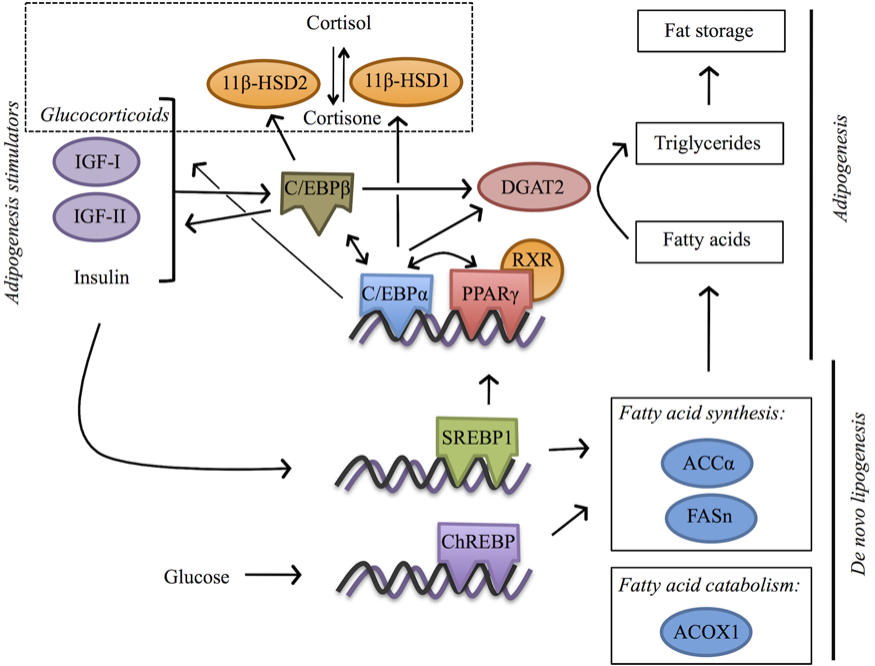
Schematic overview of the selected lipogenic genes and their function. Adipogenesis is characterized by the expression of PPARγ, C/EBP-family members and SREBP. Insulin, glucocorticoids or IGF stimulate the expression of C/EBPβ, which in turn stimulates the expression of EBPα and PPARγ. Upon stimulation, PPARγ forms a heterodimer with RXR and together with EBPα regulate the expression of genes involved in adipogenesis and lipogenesis [26]. C/EBPα and C/EBPβ also regulate the expression of 11β-HSD1 and 11β-HSD2 (which catalyse the activation and deactivation, respectively, of cortisol and regulate glucocorticoid metabolism) [40] and of IGF-I and IGF-II (which regulate growth, cell proliferation and development) [41, 42]. SREBP1 and ChREBP, are stimulated upon insulin and glucose secretion, respectively, and regulate synergistically the expression of the *de novo* lipogenesis enzymes FASn and ACCα [43] that catalyse fatty acid synthesis. SREBP1 also regulates the expression of PPARγ [44]. Fatty acid catabolism through β-oxidation in peroxisomes is catalysed by ACOX1 [45]. Excess fatty acids derived from food, break-down from stored fat or synthesized through *de novo* lipogenesis are converted to triglycerides through a pathway the last step of which is catalysed by the DGAT2 enzyme. The transcriptional regulation of DGAT2 is also regulated by EBPβ and to a lesser extent by EBPα [46].

Following the above observations, the present study was constructed to investigate the TBT-induced disorders on the lipid metabolism of males and females of the model teleost zebrafish (*Danio rerio*), following chronic exposures (9 months) to low environmentally relevant concentrations (10 and 50 ng/L as Sn). Zebrafish are recommended as test species in a number of ecotoxicological test protocols [39]. As end-points we screened the effects of TBT on the whole-body morphological parameters, the liver size and triglyceride content, as well as on the expression pattern of transcription factors and metabolizing enzymes that play a key role in the regulation of lipids (Fig 1).

**Fig 2:**
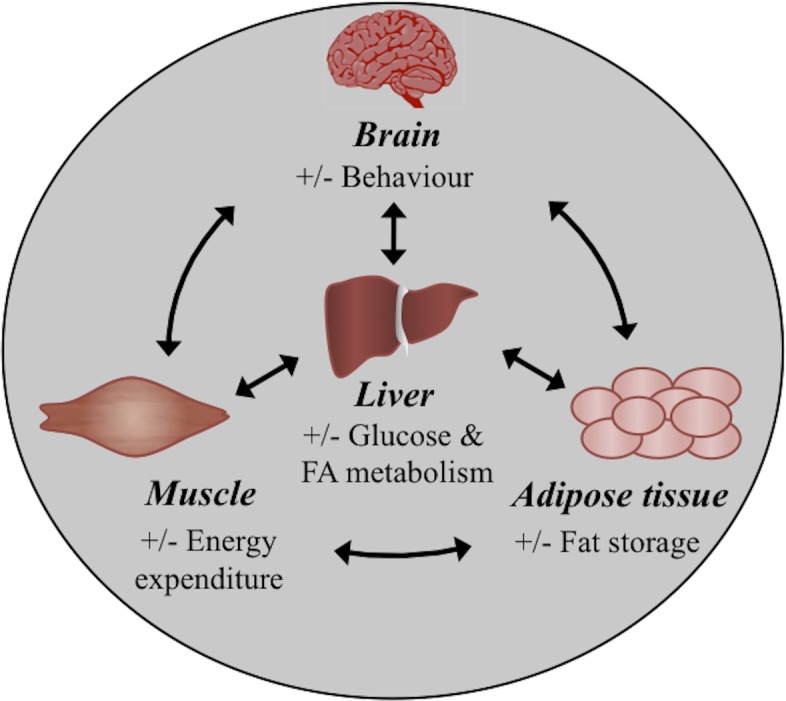
Simplified schematic overview of the function of the major organs involved in lipid homeostasis. The liver has a central role in the synthesis, metabolism and distribution of glucose and fatty acids. The adipose tissue is the principal site of excess energy storage in the form of fat (triglycerides) and liberates fatty acids upon demand. The muscle is the major site of lipid oxidation and energy expenditure. The brain acts as a lipid sensor; it receives signals from the other organs to adjust energy homeostasis by changing behaviour (e.g. food intake and expenditure). When energy consumption exceeds expenditure excess triglycerides are stored not only in adipose tissue but in muscle and liver as well, leading to metabolic disorders [48, 49].

Out of the four major organs involved in energy homeostasis (Fig 2), we selected the liver and the brain since whole-body metabolic disorders may be reflected in the metabolism in these tissues. The liver is a particularly good indicator of disease as it has a limited capacity to store fat and therefore excess synthesis and storage may lead to hepatomegaly, NAFLD, hepatocyte apoptosis and hepatic steatosis [47]. On the contrary, there is very little information on the role of the brain in the lipid metabolism of teleosts and therefore the expression of the selected lipogenic genes in the liver was also studied in the brain.

Our data show that TBT alters the body and hepatic metabolic reserves, induces the liver triglyceride content in males and perturbs the expression profile of lipogenic genes in both liver and the brain of zebrafish. We observed a clear sexual dimorphism in morphological parameters and transcriptional regulation in the liver following TBT exposure. Finally, we identified the brain lipogenic transcription profile of fish as a novel target of TBT in both males and females, which has not been investigated in mammals yet.

## Material and Methods

### Chemicals

Infinity Triglycerides Liquid Stable Reagent was purchased from ThermoScientific. Tributyltin Chloride (TBTCl; 96% purity) and all other reagents were obtained from Sigma-Aldrich unless stated otherwise in the text.

### Experimental animals- breeding

All experiments conducted in this study were carried out at Biotério de Organismos Aquáticos (BOGA, CIIMAR) aquatic animal facilities and have been approved by the CIIMAR ethical committee and by CIIMAR Managing Animal Welfare Body (ORBEA) according to the European Union Directive 2010/63/EU “on the protection of animals used for scientific purposes”. The breeding stock of adult wild-type zebrafish (*Danio rerio*; Singapore) was kept in 250 L aquaria with dechlorinated aerated water from a recirculation system equipped with mechanical and biological filters at temperature of 28 ± 1°C under a photoperiod of 14:10 h (light:dark). The fish were fed ad libitum twice a day with a commercial fish diet Tetramin (Tetra, Melle, Germany) and supplemented with live brine shrimp (*Artemia spp*.). In the afternoon before breeding, two random groups of 4–6 males and 10–12 females were transferred in net cages bottom-covered with glass marbles, placed within 30 L aquaria and fed just with live brine shrimp (*Artemia spp*.). The next morning (1.5 hour after sunlight) breeding fish were removed, the eggs were collected from the bottom of the tank by siphoning, cleaned in egg water and randomly allocated to experimental aquaria.

### Solutions preparation and exposure experiments

TBT stock solutions were prepared in acetone and four waterborne exposure conditions were set, in duplicates, in experimental aquaria: an experimental control, a solvent control (acetone at 0.0002%) and two environmentally relevant TBT concentrations (nominal concentration: 10 and 50 ng/L as Sn).

Results from our previous experiments show that continuous supply of TBT in a flow-through system fails to result in fish exposure due to adsorption of TBT to the walls of the tubes that supply the water. Hence, based on preliminary tests, we decided to dose the tanks directly, twice daily, at 0h and 8h to guarantee exposure of fish to TBT. The TBT concentration in the water was tested in the control groups and in the tanks of 50 ng/L TBT, at 20 dpf (days post fertilization) (S1 Table). In the control groups, TBT and their metabolites were below the detection limit. The analysis of organotin compounds in water was carried out according to Carvalho et al. (2007) [50].

The duration of the experiment was 9 months. Initially, 300 eggs were randomly allocated in 5 L aquaria situated within 30 L aquaria in a semi-static system with 7-hours water renewal with the exposure conditions as explained before. The number of eggs was selected considering 80% rate of viability [39]. At 15 dpf, larva fish were placed in the 30 L aquaria. Throughout the experiment, larvae fish were given the same food as their parents but adjusted according to fish development, number and size. At 40 dpf the fish density was reduced to 100 fish/aquaria and at 60 dpf to 25 fish/aquaria. The handling of fish was kept to a minimum to avoid suffering and distress and their health was monitored closely twice on a daily basis by examining spinal deformities and body wasting. Throughout the experimental period the temperature was checked daily (28 ± 1°C of temperature) and the water physicochemical parameters weekly (pH 7.7 ± 0.2; 6 ± 1 mg/L of dissolved oxygen; 376 μS/cm of conductivity; 0.08 ± 0.06 mg/L of ammonium and 0.01 ± 0.01 mg/L of nitrite). During the initial developmental period, unhatched eggs/dead larvae were detected by their opaque appearance and lack of movement and were immediately removed. Mortality was higher during the metamorphosis stage (approx. 10–18 dpf), when larvae are most vulnerable as the yolk sac is gradually depleted and the larvae must adapt to exogenous feeding. After 24 dpf mortality was practically zero and fish did not demonstrate signs of poor health. At 60 dpf the overall mortality rate along the exposure groups was between 34 and 41% (S2 Table), which is within the normal range observed in previous studies [31, 51].

At the end of the experiment, 15 male and female zebrafish were randomly selected from each of the duplicate exposure conditions and sacrificed with anaesthetic overdose using 300 mg/L 3-amino-benzoic acid ethyl ester (MS-222) to guarantee minimum pain and alleviation from discomfort, according to Annex IV of the EU Directive 2010/63/EU. After recording the morphological parameters, the liver and brain tissues of individual zebrafish were dissected and placed either in RNAlater for RNA extraction or, in the case of liver samples designated for triglyceride content analysis, at -80°C. All samples were assessed in a random order. To confirm the uptake and accumulation of TBT in fish, the remaining liver samples were processed for TBT and its degradation products dibutyltin (DBT) and monobutyltin (MBT), following lyophilisation, as described in Carvalho et al. (2007) [50] (S1 Appendix).

### RNA purification and cDNA synthesis

Total RNA was purified from either 5 mg of liver tissue or whole brain tissue using the illustra RNAspin Mini RNA Isolation Kit (GE Healthcare) and quantification was performed using the Quant-iT^TM^ RiboGreen^®^ RNA Reagent and Kit (Invitrogen). RNA quality was verified by electrophoresis in 1% agarose gels. Total cDNA was generated either from 0.5 μg or 0.15 μg of total RNA extracted from the liver or brain tissue, respectively, using the iScript^TM^ cDNA Synthesis Kit (Bio-Rad). All assays were carried out following the manufacture’s protocol.

### Real-Time PCR

Fluorescence-base quantitative (real-time) PCR was used to evaluate gene expression profiles. For each treatment and tissue, the expression of individual target genes was performed with the Mastercycle ep *realplex* system (Eppendorf). Each sample was amplified in duplicate using 96-well PCR plates in a 10 μL reaction volume containing 2.5 μL of cDNA (1:5 dilution), 2 μL HOT FIREPol Evagreen qPCR Mix Plus (no ROX; Solis BioDyne) and 200 nM of each forward and reverse primer (S3 Table). Primers were designed using the Primer-Blast-NCBI tool and synthesized by STABVIDA (Portugal). In each plate, a ‘‘no template control” was included to determine the specificity of target cDNA amplification. The three-step real-time PCR program consisted of an enzyme activation step at 95°C (10 min), a 40-cycles amplification step of denaturation at 95°C (10 s), annealing at 60–64°C depending on target gene (30 s) and extension at 72°C (30 s), and a melting curve step generated at the end of every run to confirm specificity of the assays. The PCR products were analyzed by agarose gel electrophoresis to confirm the presence of single bands. Reference genes [ribosomal protein l8 (rpl8) for liver samples and β-actin for brain samples] were selected according to their stability across the exposure groups and controls in each tissue, following validation by one-way ANOVA. The PCR efficiency for target genes of interest and the reference genes was determined through a standard curve, using serial dilutions of cDNA pools from each tissue. Only genes with efficiencies between 88–109% were used. The expression of all genes was measured from cDNAs belonging to the same fish, allowing for comparisons among genes in the same individual.

Relative quantification of target genes was performed according to the Pfaffl method [52]. The relative gene expression ratio (*R*) of a target gene was calculated based on the gene efficiency *E* and the ∆Ct between the sum of the control group and the unknown sample, and expressed in comparison to the reference gene: R = (E_target_)^∆Ct target (∑Control-sample)^/ (E_ref_) ^∆Ct reference (∑Control-sample)^

Data were then expressed as fold changes of the solvent control group (value of treatment/mean value of solvent control group).

### Lipid extraction and triglyceride determination.

Lipids were extracted from the liver tissues of sacrificed animals using a low toxicity solvent protocol, as described by Schwartz and Wolins [53]. Extracts were re-suspended in isopropanol and triglyceride content was measured using Infinity Triglycerides Liquid Stable Reagent (Thermo Scientific), according to manufacture’s protocol. Samples were measured in duplicates and absorbance was determined at 540 nm using a microplate reader (PowerWave 340, Bio-Tek). Triglycerides concentration was calculated against a standard curve generated from triolein standards. The intra-assay variability, calculated by determining the coefficient of variation (CV) for five replicates of a pooled sample was 8.7%.

### Statistical Analysis

Fulton’s condition factor (CF) was calculated according to the equation CF = (Body Weight × Length^-3^) × 100. The hepatosomatic index (HSI) was calculated as (Liver Weight / Body Weight) × 100. Results are mean values ± SEM. Control and solvent control groups were compared using Student’s *t*-test to verify that there were no significant differences and thereafter the latter was used for comparison against the exposure groups. Normality and homogeneity of variance was confirmed using Shapiro-Wilk and Levene’s tests, respectively.

Thereafter, all data were analyzed by one-way ANOVA followed by Dunnett's *post hoc* test. *P*-values of less than or equal to 0.05 were considered statistically significant unless stated otherwise. For morphological parameters and hepatic triglyceride levels, when parametric assumptions were not fulfilled, data was log-transformed. In the case of qPCR gene fold expressions, all data was previously log-transformed (log_2_) to stabilize the variance. In very few cases, transformed data did not meet the assumptions for parametric testing, thus data were analyzed using a Kruskal-Wallis followed by Mann-Whiney U test with Bonferroni adjustment.

Discrimination of gene expression profiles associated with TBT exposures in males and females was achieved by statistical analysis using Principal Component Analysis (PCA). PCA involves a mathematical procedure that transforms a number of possibly correlated variables into a smaller number of uncorrelated variables called “principal components” (PCs). PCA was applied to the log-transformed ratios (exp/cont) of all genes, within each tissue. Thereafter, we performed one-way ANOVA followed by Dunnett's comparison *post hoc* test with PCs as dependent variables and exposure groups as independent variables to test which treatments were discriminated significantly within each PC (*p*<0.05). Statistical analysis was performed using the software package SPSS/Mac version 20 and “R”.

## Results

### Physiological Parameters

Table 1 shows the mean weight, length, condition factor (CF) and hepatosomatic index (HSI) of zebrafish (*Danio rerio*), following 9-month chronic waterborne exposure to 10 and 50 ng TBT/L (as Sn). In TBT-exposed fish, the body weight increased significantly only in male fish exposed to 10 ng/L TBT by 10% compared to the control (*p* = 0.027) and marginally (8%) in male fish exposed to 50 ng/L TBT (*p* = 0.095). The CF, which could be considered the fish equivalent to the body-mass index in humans, was altered in both exposed males and females but in an opposite manner. Thus, CF showed an 11% increase in males at 10 ng/L (*p* = 0.001), whereas in females it showed an 11 and 12% decrease at 10 (*p* = 0.006) and 50 ng/L (*p* = 0.002) exposure, respectively. Exposed females revealed hepatomegaly as indicated by the increase in the HSI by 33 and 40% at 10 (*p* = 0.018) and 50 ng/L (*p* = 0.004) exposure, respectively. No significant differences among treatments were found in fish length.

**Table 1:** Morphological parameters of male and female zebrafish following chronic waterborne exposure (9 months) to tributyltin (TBT). Values are mean±SEM (n = 15); CF and HSI are the condition factor and hepatosomatic index, respectively; **p<0.05 and *<0.1 (compared to solvent control; one-way ANOVA followed by Dunnett's test)

TBT (ng Sn/L)	Weight (g)	Length (mm)	CF	HSI
*Males*				
Control	0.69±0.02	42.14±0.48	0.92±0.02	1.26±0.17
Solvent control	0.68±0.01	42.56±0.53	0.89±0.03	1.33±0.21
10	0.75±0.02**	42.65±0.33	0.97±0.02**	1.57±0.15
50	0.74±0.02*	43.22±0.32	0.91±0.02	1.45±0.16
*Females*				
Control	1.12±0.07	44.36±0.80	1.29±0.04	2.35±0.21
Solvent control	1.10±0.04	43.81±0.45	1.31±0.04	2.66±0.19
10	1.05±0.04	44.79±0.45	1.17±0.03**	3.53±0.27**
50	1.07±0.06	45.10±0.69	1.15±0.03**	3.72±0.21**

### Hepatic Triglyceride content

**Fig 3:**
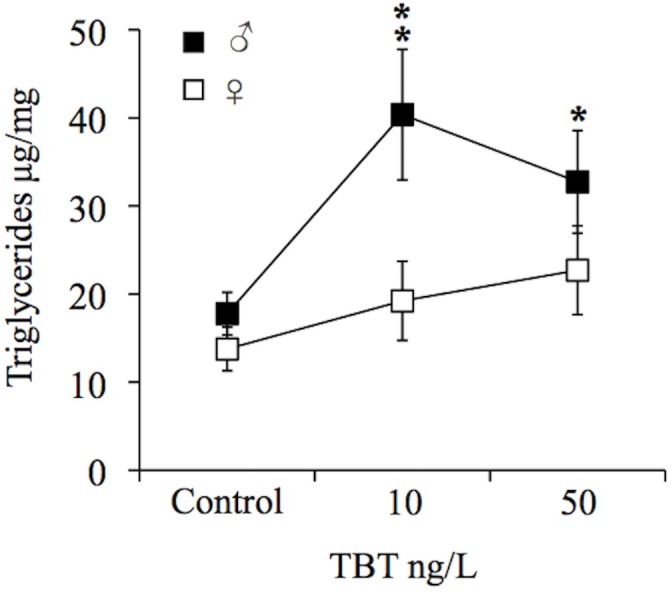
TBT effects on hepatic triglycerides. The effect of TBT on hepatic triglyceride levels in male and female zebrafish following chronic exposure (9 months) to waterborne 10 and 50 ng/L of TBT (as Sn). Values as mean ± SEM (n = 4). * *p<0.05, *p<0.1 compared to control (one-way ANOVA, followed by Dunnett’s test).

Next we examined whether the increase in liver size was due to an increase in triglyceride content. TBT exposure caused a significant increase in the hepatic triglyceride levels in male fish, of approximately 2.2-fold at 10 (*p* = 0.042) and a marginal increase of 1.8-fold at 50 ng/L (*p* = 0.086) exposure (Fig 3). Hepatic triglyceride levels also increased in females, however not being significantly different from the control.

### Gene expression

Mammalian studies indicate that TBT exposure induces fat accumulation *in vivo* through the transcriptional regulation of lipogenic and adipogenic genes [17, 19]. Thus, we next selected to study the effects of TBT exposure on the transcription level of genes involved in adipogenesis [RXRα/a, PPARγ, CCAAT-enhancer-binding protein alpha (C/EBPα) and beta (C/EBPβ), diacylglycerol O-acyltransferase 2 (DGAT2)], lipogenesis [sterol regulatory element-binding protein 1 (SREBP1), carbohydrate-responsive element-binding protein (ChREBP), acetyl- CoA carboxylase alpha (ACCα), fatty acid synthase (FASn), acetyl-CoA oxidase 1 (ACOX1)] and also in glucocorticoid metabolism [11beta-hydroxysteroid dehydrogenase 2 (11β-HSD2) and 3 alpha (11β-HSD3α)], growth and development [insulin-like growth factor-I (IGF-I) and II alpha (IGF-IIα)] (Fig 1) in the liver and the brain of zebrafish.

#### Liver

**Fig 4:**
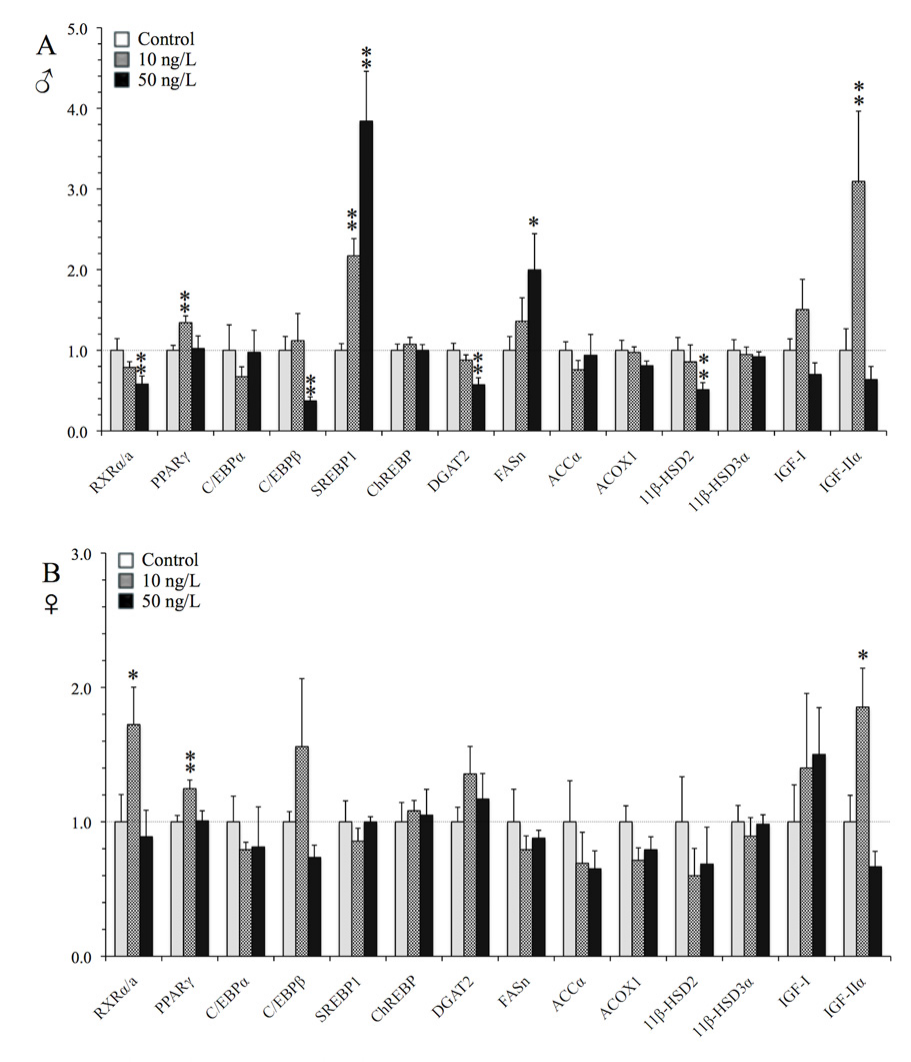
TBT *in vivo* induction of lipogenic genes in the liver. qRT-PCR analysis of selected lipogenic transcription factors and metabolizing enzymes in the liver of (A) male, and (B) female zebrafish following chronic exposure (9 months) to waterborne 10 and 50 ng/L of TBT (as Sn). Values were normalized to rpl8 and expressed as the average fold changes ± SEM (n = 8) of the solvent control group. **p<0.05 and *p<0.1 compared to solvent control (one-way ANOVA, followed by Dunnett’ test).

In the liver, chronic exposure of male and female zebrafish to the low (10 ng/L) TBT concentration up-regulated the expression of PPARγ–the “master” regulator of adipogenesis [26] -by 34% in males (p = 0.012) and 25% in females (p = 0.039) and IGF-IIα—the regulator of growth and development in vertebrates [54] -by approximately 3-fold in males (p = 0.025) and 2-fold in females (p = 0.064) (Fig 4A and 4B). Thereafter, males, which had significantly higher hepatic triglyceride levels, showed induced de novo lipogenesis responses as indicated from the up-regulation of SREBP1 (approximately 2- and 4-fold at 10 and 50 ng/L, p = 0.001 and p<0.001, respectively) and FASn transcription (2-fold at 50 ng/L, p = 0.085), two key lipogenic genes that are also highly expressed during adipogenesis [44]. In parallel, at 50 ng/L exposure, 11β-HSD2- the enzyme that deactivates cortisol- was down-regulated by 48% (p = 0.037) [55]. Thereafter, TBT suppressed the mRNA levels of both the adipogenic markers C/EBPβ and DGAT2 [46] in male livers by 43% (p = 0.002 and p<0.001, respectively). The transcription of RXRα/a, the heterodimeric partner of PPARγ, was altered in a gender- and dose-specific manner in the liver, being significantly down-regulated in males at 50 ng/L TBT exposure (by 42%, p = 0.016) and significantly up-regulated in females at 10 ng/L (by 72%, p = 0.043). In the case of females, which exhibited hepatomegaly, we only observed subtle adipogenic responses in the transcription of genes, where apart from RXRα/a, PPARγ and IGF-IIα, the expression of the adipogenic genes C/EBPβ and DGAT2 showed a tendency to increase at the low TBT exposure, however not being statistically significant (p>0.1).

#### Brain

**Fig 5:**
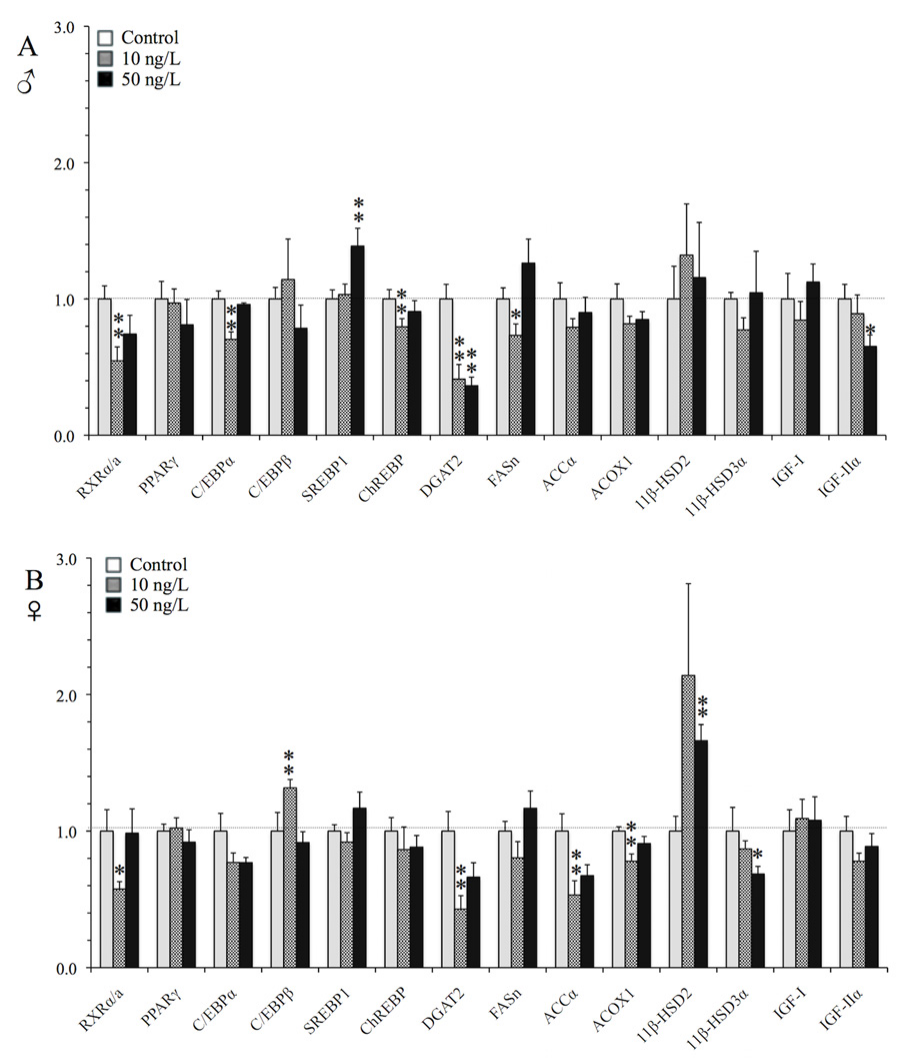
TBT *in vivo* induction of lipogenic genes in the brain. qRT-PCR analysis of selected lipogenic transcription factors and metabolizing enzymes in the brain of (A) male, and (B) female zebrafish following chronic exposure (9 months) to waterborne 10 and 50 ng/L of TBT (as Sn). Values were normalized to β-actin and expressed as the average fold changes ± SEM (n = 7) of the solvent control group. **p<0.05 and *p<0.1 compared to solvent control (one-way ANOVA, followed by Dunnett’s test).

In the brain, although the transcription of PPARγ remained unchanged following TBT exposure, RXRα/a was significantly down-regulated in both genders at the low TBT exposure (10 ng/L; by 45% in males and 43% in females; p<0.008 and 0.058 respectively) and so did the expression of the adipogenic factor DGAT2 (by 59% in males and 58% in females, p = 0.001 and p = 0.006 respectively; Fig 5A and 5B) as well as C/EBPα but the down-regulation was only significant in males (30%, p = 0.014). Overall in males, responses were dose-specific. The transcription of ChREBP, which controls synergistically with SREBP1 the expression of lipogenic enzymes FASn and ACCα [43], was marginally down-regulated by 20% at 10 ng/L TBT exposure (p = 0.070), and so did FASn (by 27%, p = 0.069), whereas at 50 ng/L exposure the lipogenic factor SREBP1 was up-regulated significantly by 39% (p = 0.011), and its encoding enzyme FASn showed a tendency to increase without being statistically significant (p = 0.297). At 50 ng/L exposure, DGAT2 was down-regulated by 64% (p<0.001) and so did IGF-IIα (by 45%, p = 0.078). In the brain of females, at 10 ng/L exposure TBT up-regulated the expression of the adipogenic regulating factor EBPβ but down-regulated the expression of ACCα (47%, p = 0.009) and ACOX1 (26%, p = 0.008). At 50 ng/L exposure, TBT up-regulated the cortisone synthesizing enzyme 11β-HSD2 by approximately 2-fold (p = 0.012), whereas 11β-HSD3α, the cortisol synthesizing enzyme, was marginally down-regulated (p = 0.098).

### PCA analysis of the gene expression profiles of males and females in the liver or brain.

A PCA analysis was performed on the gene expression data to highlight the differences in the transcription profile of lipogenic transcription factors and energy metabolism enzymes among treatment groups in either liver or brain tissues of male and female zebrafish. For each of the liver and brain gene expression data, 5 components had eigenvalues over Kaiser’s criterion of 1, which explained together 69.7% and 70.4% of the total variance in the data for liver and brain tissue, respectively (S4 and S5 Tables).

**Fig 6:**
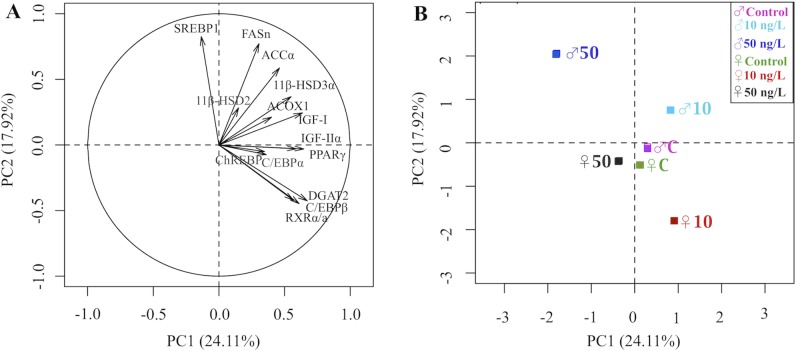
Principal Component Analysis (PCA) in the liver. PCA applied to the gene expression data of the lipogenic transcription factors and metabolizing enzymes in the liver of male and female zebrafish exposed to waterborne 10 and 50 ng/L of TBT (as Sn) for 9 months. Data are presented against PC1 and PC2 as (A) a loading plot of the 14 studied genes and (B) a scores’ plot of each treatment group of males and females separately.

In the liver, PC1 and PC2, which explain together 42% of the total variance, discriminated significantly the high TBT exposure group of males from the control, whereas only PC2 discriminated marginally the low TBT exposure group of females, showing an opposite response than males (Fig 6A and 6B; S4 Table). The two clusters of genes that separated the genders along PC2 (SREBP1-FASn-ACCα and RXRα/a-C/EBPβ-DGAT2) are in agreement with adipocyte studies showing that SREBP1 regulates the transcription of FASn and ACCα [44], and C/EBPβ of DGAT2 [46].

**Fig 7:**
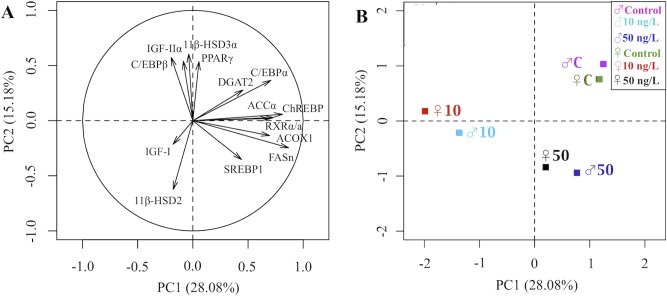
Principal Component Analysis (PCA) in the brain. PCA applied to the gene expression data of the lipogenic transcription factors and metabolizing enzymes in the brain of male and female zebrafish exposed to waterborne 10 and 50 ng/L of TBT (as Sn) for 9 months. Data are presented against PC1 and PC2 as (A) a loadings plot of the 14 studied genes and (B) a scores’ plot of each treatment group of males and females separately.

In the brain, even though the changes in the expression of the some genes were less evident, PCA analysis revealed that the transcriptional profile was very similar between sexes. Thus, PC1 and PC2, which explained together 43.3% of the total variance, separated significantly the low TBT exposure group and the high TBT exposure group from the control group, respectively, in both genders (Fig 7A and 7B; S5 Table). Here, the low TBT exposure groups showed a negative significant correlation with PC1 and thus with RXRα/a, DGAT2, FASn, C/EBPα, ACOX1, ACCα, SREBP1 and ChREBP, whereas the high TBT exposure groups showed a negative significant correlation with PC2 and consequently with PPARγ, C/EBPβ, IGF-II, 11β-HSD3α and C/EBPα and a positive correlation with SREBP1, FASn and 11β-HSD2 (Fig 7A and 7B; S5 Table).

## Discussion

The lipid metabolism as a target of EDCs is a recent field in endocrine disruption research. Studies on mammalian species demonstrate that specific contaminants induce fat accumulation, particularly when exposure takes place during sensitive life-time windows, such as embryogenesis and the early stages of development [1, 2, 6]. Fish are exposed to EDCs in their natural environment throughout their life-cycle and although several lipid regulatory mechanisms are conserved between fish and mammals [32, 33], there are very few studies on the interaction of these compounds with fish lipogenesis. In this context, the present work demonstrates that the mammalian “*obesogen*” TBT -reported to increase adipose mass and induce NAFLD in mammals through the activation of PPARγ and RXR [17–19]- perturbs the energy reserves and transcriptional regulation of lipid metabolism in the model teleost zebrafish following chronic exposure to very low and environmentally relevant concentrations. This is of concern, taking into account that lipids have a fundamental role in metabolism, growth, reproduction and migration of fish [56]. Therefore, any alterations on their homeostasis may result in adverse effects at individual and population level.

Exposure of zebrafish to TBT since the pre-hatch stage revealed sex-dependent differences on the somatic energy reserves of fish when adults: an increase in body weight and condition factor in males and suppressed condition factor in females. There is very limited information on the effects of TBT on the weight control mechanisms in fish. Recently, Meador and colleagues [57] found an increase in the body weight of juvenile salmon (*Oncorhynchus tshawytscha*) fed with low-doses of TBT (0.4–3.5 ng/g fish/day) for 62 days, whereas fish that received the highest TBT dose (458 ng/g fish/day) showed reduced body weight. In a different study, dietary exposure of genetically female flounder (*Paralichthys olivaceus*) for 65 dpf to 0.1 and 1 μg TBT/g *ad libitum*, daily, resulted in reduced body weight, total length and consequently condition factor [58]. Differences in body weight between genders following exposure to TBT have been observed in mammalian studies, where reduced body weight in female rats [59], body weight gain in male mice [18, 19], dose-relevant alterations in body weight of male mice [60], or no alterations at all [17] have been reported. These results, in combination with those of the present study, show that TBT alters the overall weight and somatic energy reserves in vertebrates, in a species-, dose- and gender-dependent manner.

Exposure to TBT lead to an increase in HSI and hepatic triglyceride levels, with exposed males showing significant triglyceride accumulation and females of hepatomegaly, which suggest hepatic metabolic dysfunction. We also noticed an increased adipose mass in zebrafish viscera (S1 Fig). Similar to our results, an increase in lipid-containing vacuoles in fish hepatocytes following exposure to TBT has been reported before, but in fish that were exposed during adulthood either to higher TBT concentrations [61] or for a much smaller period of time [62]. The capacity of TBT to induce lipid accumulation in fish is not restricted to the liver. Juvenile Chinook salmon fed TBT for 57 days showed increased whole body total lipids together with triglyceride levels at 150 ng/g exposure [57] and immature males and females of rockfish exposed to 100 ng/L TBT (as Sn) for 48 days showed increased total lipids and lipid droplets in the testis [63] or ovary [64]. These data suggest that the mammalian *obesogen* TBT promotes lipid accumulation in fish and as the present study shows, even at concentrations as low as 10 ng/L. Hepatomegaly in fish usually indicates the presence of lipid-filled hepatocytes [65] and in some cases storage of excess glucose in the form of glycogen [66]. Even though glycogen levels were not measured in the present study, the observation that triglycerides levels increased significantly only in males, suggests that the enlargement of female liver may be due to increased glycogen content.

**Fig 8:**
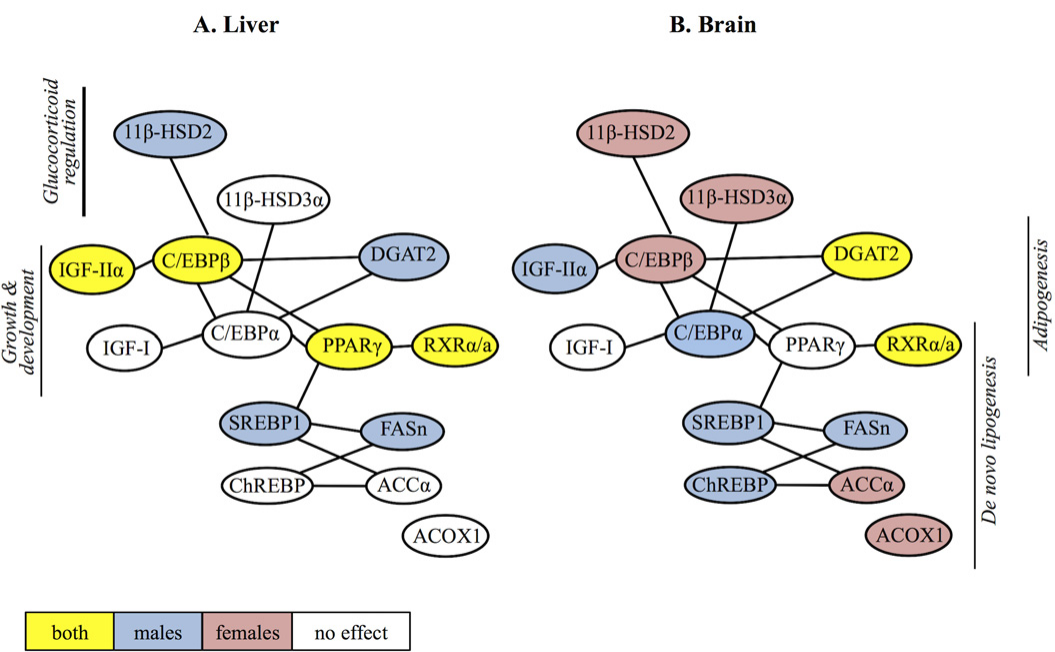
Summary of the TBT-altered genes in the liver and brain of zebrafish. Schematic representation summary of genes that were either up- or down-regulated (p<0.1) in males (blue), females (pink), both genders (yellow) or neither (blank) in (A) the liver and (B) the brain of zebrafish following chronic waterborne exposure (9 months) to either 10 or 50 ng/L TBT (as Sn). The connectors indicate the genes that are related.

At the molecular level, the metabolic regulation of lipids is controlled by the transcription of factors, which regulate the expression of genes coding for enzymes involved in the lipid synthesis and metabolism [67]. Here we show that the observed TBT-induced somatic and hepatic effects occurred in parallel with significant alterations in the expression levels of transcription factors and enzymes in the liver and the brain, that regulate different pathways of the lipid metabolism (Fig 8A and 8B). The activation of transcription factors leads to gene transcription and synthesis of the corresponding enzymes. Thereafter the transcription of the nuclear receptor itself is controlled by phosphorylation events by specific kinases, which determine its up-regulation or down-regulation [68]. Thus, the observed alterations in the expression of the nuclear receptors in the present study indicate that TBT interacts with these receptors. We also observed a nonlinear dose-response relationship in the expression of most of the selected lipogenic genes in female liver and in the brain of both genders, showing a U- or inverted U-shaped response, where the low TBT exposure appeared more potent than the higher exposure. This is a common pattern in endocrine studies, especially related to nuclear receptor activation during chronic exposure experiments. A low concentration exposure to an endocrine disruptor may result in the activation of a specific receptor and produce an endocrine response. However, at higher doses or prolonged exposure, the receptor gets saturated and no further responses are observed or the receptor may even be down-regulated to achieve homeostasis [69]. At even higher exposure concentrations theses responses may be totally suppressed or compensated, a phenomenon also known as “hormesis” [70].

The effects of such low concentrations of TBT on the transcription of lipid regulating genes in the liver of fish combined with the increased triglyceride levels or hepatomegaly, in males and females respectively, is of concern. In fish, mammalian-like liver dysfunction diseases such as NAFLD and liver steatosis may occur [35, 36], which lead to an increased oxidative damage and cell death [71]. In our study, both TBT exposures caused a significant up-regulation of SREBP1 and FASn genes in the liver of males, who also contained high hepatic triglycerides levels. An up-regulation of the *de novo* lipogenesis genes SREBP1 and FASn has been observed in the liver of zebrafish larvae, either following exposure to ethanol [65] or by over-expression of the cannabinoid receptor [72]. This was accompanied by the development of hepatic steatosis and fatty liver, suggesting that TBT exposed male fish in our study experience signs of NAFLD. In the case of severe hepatic metabolic conditions manifested in transgenic zebrafish either by specific expression of Hepatitis virus X protein [35] or over-expression of the *Ying-Yang 1* transcription factor [36], fish develop hepatic steatosis, fatty liver and hepatocyte inflammation resulting in an increased death rate. In parallel, the transcription of lipogenic factors (e.g. PPARγ, C/EBPα, SREBP1) and enzymes (e.g. FASn, ACCα, DGAT2) in the liver is significantly up-regulated. In female zebrafish of our study, the low TBT exposure (10 ng/L) up-regulated the expression of PPARγ and RXRα/a in the liver, which showed a positive correlation with C/EBPβ and DGAT2 (S4 Table). Although the hepatic lipid accumulation in females was not significant, these gene expression responses indicate signs of adipogenesis stimulation. Moreover, in male fish exposed to 50 ng/L TBT (as Sn), the increase in the up-regulation of the *de novo* lipogenesis genes SREBP1 and FASn didn’t result in the production of more triglycerides. Interestingly, these adipogenic genes C/EBPβ and DGAT2, as well as RXRα/a, were down-regulated. Overall, transcriptional alterations in the liver reflect hepatic metabolic dysfunction in fish as observed in mammals, and therefore the alterations observed in the present study, although less severe, suggest that fish experience lipid deregulation, which may lead to disease.

The “*obesogenic*” effects of TBT have been studied in mammals, demonstrating that TBT exposure during prenatal or early postnatal stages causes an increase in adipose mass and liver lipids [17–19]. Similar to our findings, single injections of adult mice with TBT or *in utero* exposure regulated the transcription of both adipogenic (PPARγ and its target genes) and lipogenic genes (SREBP1, FASn, ACCα, C/EBPα,β) in the liver and bone marrow MSCs [19] as well as testis and adipose tissue [17]. The results were comparable to the effects of the synthetic RXR ligand AGN195203 and PPARγ ligands troglitazone [17] or Rosiglitazone (Rosi) [19]. These data, together with the observations that TBT is a high affinity ligand of RXRα and PPARγ [17, 24, 25], it induces adipocyte differentiation *in vitro* [17, 24, 27, 28] and that TBT-induced effects are blocked by PPARγ antagonists GW9662 or T0070907, totally [27] or partially [28] strongly suggest that TBT induces adipogenesis, both *in vitro* and *in vivo* through the transcriptional activation of RXR:PPARγ heterodimer.

In fish RXR and PPARγ show a high similarity with the human or mouse orthologues [37, 38]. In the present study, although the protein levels were not quantified, we show that TBT induced an increase in triglyceride levels or hepatomegaly and altered the transcription of RXRα/a, PPARγ and genes downstream the RXR and PPARγ pathways. Our data are in agreement with other studies, showing that TBT may interfere with the expression of RXR and/or PPARs in liver or other tissues of fish [63, 73, 74]. The interaction of TBT with either RXR or PPARγ is an ongoing research. In a recent study, TBT induced lipid accumulation in zebrafish larvae, but failed to activate zebrafish PPARγ *in vitro* [75]. A different study showed that exposure of zebrafish larvae to either TBT or the PPARγ synthetic ligand Rosi for 24 hours, induced comparable lipid accumulation [76]. Given that the RXR:PPARγ heterodimer is permissive, being activated by both PPARγ and RXR ligands, it may be hypothesized that PPARγ and lipid induction in zebrafish under chronic TBT exposure occurs through RXR [75].

A novel finding of the current work relates with the perturbation of the transcription profile of key genes involved in lipid homeostasis in the brain of fish following TBT exposure, an observation that has yet to be investigated in mammals. This is of concern since the central nervous system regulates the whole body lipid homeostasis: it acts as an energy sensor by detecting lipid molecules and hormones secreted from other tissues and regulates food intake and expenditure in response [48]. Thus, triglycerides administration in the brain of rats stimulates insulin secretion and down-regulates hepatic glucose production [77] and induction of obesity-related hormones such as leptin and ghrelin in the brain repress or stimulate, respectively, the transcription of enzymes involved in FA *de novo* synthesis in the liver and adipose tissue [78]. In relation to the genes studied here, enzymes of the fatty acid synthesis pathway such as FASn and ACC are highly expressed in the brain of rats and their regulation has been attributed to feeding behaviour. Inhibition of FASn or activation of ACCα result in reduced food intake and body weight loss [79, 80]. Fish share several signaling pathways of brain metabolism with mammals [81]. The fish brain uses glucose and ketone bodies as fuels and has the capacity to synthesize and metabolize fatty acids [81]. Moreover, the mammalian appetite regulating factors leptin and ghrelin, are all expressed in adult fish brain [82, 83]. Thus, the observed TBT alterations in the brain transcription profile of zebrafish are signs of a central deregulation of the lipid metabolism. Further studies should focus on the relation of these alterations to the physiological responses at the whole organism level.

Despite the *obesogenic* effects of TBT, very few studies have examined its interaction with glucocorticoid regulation and IGFs transcription, even though both glucocorticoids and IGF-I induce adipogenic responses and promote preadipocyte maturation [41]. In teleosts, IGF-I and -II play a key role in growth and development [54] and 11β-HSDs regulate the cortisol levels, the key hormone released during stress response and controls the hypothalamus–pituitary–interrenal axis [55]. Both the IGF system and 11β-HSDs are potential targets of environmental contaminants in fish [84, 85]. Here, the transcription of IGF-IIα and 11β-HSD2s was altered by TBT exposure in either liver or brain of zebrafish, with IGF-IIα being strongly up-regulated in both male and female livers from the low exposure group along with the induced hepatic triglyceride or HSI in males and females, respectively. In females, we hypothesize that the gene expression of 11β-HSDs in the brain at high TBT exposure (50 ng/L) was inversely regulated to suppress cortisol levels. Cortisol in fish activates glucose synthesis and glycogenolysis processes [86] and therefore, changes in the expression of 11β-HSDs may alter the glycogen reserves. Whether the effects of TBT on 11β-HSDs signalling induced glycogen storage in the liver of females that exhibited hepatomegaly should be further investigated. Moreover, in rainbow trout the IGF-IIα promoter region has C/EBPβ responsive elements [42] and C/EBPβ regulates 11β-HSD2 transcription in human cell lines [40]. Interestingly, in our study C/EBPβ was correlated with 11β-HSD2 and ΙGF-IIα in fish liver (S4 Table), and with IGF-IIα in fish brain (S5 Table), suggesting that C/EBPβ may be a prime target of TBT. The interplay between TBT-induced changes in IGF-II and 11β-HSD2 signalling and lipid or glycogen accumulation in fish is a subject that requires further research.

The observed gender-specific differences in the transcription profile of zebrafish in the liver are not surprising. Similar to our results, the expression of lipogenic genes in mice showed a clear sexual dimorphism in the liver, which was less evident in the brain [87]. Some modest gender-dependent effects in the transcription of lipogenic genes were also observed in mice exposed to TBT *in utero*, with females showing stronger signs of the NAFLD-like phenotype than males [51]. Zebrafish also show a sexual dimorphism in the lipogenic hepatic gene transcription profiles [88]. In the present study the regulation of most lipogenic genes in the liver was gender-specific, with males showing stronger lipid synthesis responses (SERBP1 and FASn up-regulation). Gender-specific effects on lipid metabolism were also observed in zebrafish exposed to the endocrine disruptor Perfuorononanoic acid for 3 months; males showed higher hepatic triglyceride levels and up-regulation of hepatic PPARs, C/EBPs and fatty acid binding protein (fabp) mRNA levels, whereas exactly the opposite was true for females [89]. This sexual dimorphism suggests that genders should be evaluated separately, not only when studying EDCs’ effects on reproduction but also on lipid metabolism. The observed sex-dependent alterations do not seem to be related to differential TBT uptake and accumulation in the liver of fish. Analysis of the remaining liver samples for TBT and its break-down products DBT and MBT, showed similar organotin levels between genders (S6 Table). In fact, the liver revealed higher levels of DBT than TBT. The rapid metabolism of TBT in fish liver has been reported before and it’s not observed in the brain or other organs [90]. The differences in the accumulation of organotins between tissues may play a role in the different molecular responses observed in the liver and brain of zebrafish. For example, a recent study revealed that DBT and not TBT acts as a potent antagonist of the glucocorticoid receptor in human cell lines [91] and suppresses glucocorticoid signaling in zebrafish embryo [92]. Whether the observed differential response of 11β-HSDs expression in the liver and brain of zebrafish in the present study is attributed to the higher levels of DBT in the liver should be further investigated.

## Conclusions

Overall, the present study shows that the mammalian *obesogen* TBT disrupts the lipid metabolism in zebrafish following chronic exposure since the pre-hatched stage to low, environmentally relevant concentrations. The screening of the transcription profile of lipogenic genes revealed that not only the liver but also the brain is affected, a previously unidentified target of this compound. Further, we found a sexual dimorphism mainly in the morphological parameters and hepatic gene profile, which indicates that the effects of such chemicals should be studied in a gender-specific manner. Considering lipid metabolism similarities between fish and mammals and the disruption of energy balance mechanisms by TBT, future studies should investigate the lipid metabolism of fish as a target of environmental contaminants. Furthermore, the use of fish models to unravel the mechanisms underlying metabolic disorders should become a promising and informative approach.

## Supporting Information

S1 ARRIVE Checklist (PDF)

S1 Appendix Quantification of organotins in zebrafish liver. (PDF)

S1 Table TBT concentration in the water. Evaluation of the concentration of Tributyltin (TBT), Dibutyltin (DBT) and Monobutyltin (MBT) in the water from the 50 ng/L TBT (as Sn) exposure tanks at 20 days post fertilization. The aquaria were dosed twice daily (at 0 and 8 hours).
(PDF)

S2 Table Zebrafish mortality. Mortality rate in zebrafish exposed to TBT (as Sn) at 60 days post fertilization (dpf).
(PDF)

S3 Table Primer pair sequences used for RT-qPCR analysis. (PDF)

S4 Table Principal Component Analysis of the liver gene expression profile in TBT-exposed zebrafish. (PDF)

S5 Table Principal Component Analysis of the brain gene expression profile in TBT-exposed zebrafish. (PDF)

S6 Table Hepatic organotin levels. (PDF)

S1 Fig Visceral fat in TBT exposed zebrafish. (PDF)
