# Supplementary material for: The Mammalian “Obesogen” Tributyltin Targets Hepatic Triglyceride Accumulation and the Transcriptional Regulation of Lipid Metabolism in the Liver and Brain of Zebrafish
Source: PLoS One. 2015 Dec 3;10(12):e0143911. doi: 10.1371/journal.pone.0143911 (PMC4669123; doi:10.1371/journal.pone.0143911)
Supplement: S1 Appendix — (PDF) [file pone.0143911.s002.pdf]

S1 Appendix. Quantification of organotins in zebrafish liver.

Following hepatic triglyceride content quantification, the remaining liver tissues of zebrafish per treatment and gender were lyophilized and pooled together to achieve a total weight of 0.05g. Samples were then extracted with 2ml solution of 1:1 (v/v) 6M HCl/ethanol in a static ultrasonic bath for 2h and 50 μl of the supernatant was brought to a final volume of 10 ml and analysed by headspace SPME-GC-MS/MS for tributyltine and its metabolites dibutyltin (DBT) and monobutyltin (MBT) as described in Carvalho et al. (2007) [48].
