# Supplementary material for: The Mammalian “Obesogen” Tributyltin Targets Hepatic Triglyceride Accumulation and the Transcriptional Regulation of Lipid Metabolism in the Liver and Brain of Zebrafish
Source: PLoS One. 2015 Dec 3;10(12):e0143911. doi: 10.1371/journal.pone.0143911 (PMC4669123; doi:10.1371/journal.pone.0143911)
Supplement: S1 Table — Evaluation of the concentration of Tributyltin (TBT), Dibutyltin (DBT) and Monobutyltin (MBT) in the water from the 50 ng/L TBT (as Sn) exposure tanks at 20 days post fertilization. The aquaria were dosed twice daily (at 0 and 8 hours). (PDF) [file pone.0143911.s003.pdf]

S1 Table. TBT concentration in the water. Evaluation of the concentration of Tributyltin (TBT), Dibutyltin (DBT) and Monobutyltin (MBT) in the water from the 50 ng/L TBT (as Sn) exposure tanks at 20 days post fertilization. The aquaria were dosed twice daily (at 0 and 8 hours).

Sampling time (hours)	MBT (ng/L)	DBT (ng/L)	TBT (ng/L)
0h	n.d.	n.d.	37.2±6.5
8h	6.8±3.5	n.d.	8.1±6.1
24h	26.8±13.4	n.d.	3.1±1.1

Values as mean±SEM (n=2). 0h: 15 minutes after the first dosing; 8h: just before the second dosing; 24h: before the first next-day dosing. Detection limits (ng/L as Sn): MBT (3.4); DBT (2.5); TBT (2.0)
