# Supplementary material for: The Mammalian “Obesogen” Tributyltin Targets Hepatic Triglyceride Accumulation and the Transcriptional Regulation of Lipid Metabolism in the Liver and Brain of Zebrafish
Source: PLoS One. 2015 Dec 3;10(12):e0143911. doi: 10.1371/journal.pone.0143911 (PMC4669123; doi:10.1371/journal.pone.0143911)
Supplement: S2 Table — Mortality rate in zebrafish exposed to TBT (as Sn) at 60 days post fertilization (dpf). (PDF) [file pone.0143911.s004.pdf]

S2 Table. Zebrafish mortality. Mortality rate in zebrafish exposed to TBT (as Sn) at 60 days post fertilization (dpf).

Exposure	Mortality (%)
Control	34.5
Solvent control	28.7
TBT 10 ng/L	33.8
TBT 50 ng/L	41.2
