# Supplementary material for: The Mammalian “Obesogen” Tributyltin Targets Hepatic Triglyceride Accumulation and the Transcriptional Regulation of Lipid Metabolism in the Liver and Brain of Zebrafish
Source: PLoS One. 2015 Dec 3;10(12):e0143911. doi: 10.1371/journal.pone.0143911 (PMC4669123; doi:10.1371/journal.pone.0143911)
Supplement: S3 Table — (PDF) [file pone.0143911.s005.pdf]

S3 Table. Primer pair sequences used for RT-qPCR analysis.

Gene	Genbank access #	Forward primer (5´- 3´)	Reverse primer (5´- 3´)
RXRα/a	NM_001161551	ATTCAATGGC´ATCTCCTG	GCGGCTTAATATCCTCTG
PPARγ	NM_131467	GGTTTCATTACGGCGTTCAC	TGGTTCACGTCACTGGAGAA
C/EBPα	NM_131885	AACGGAGCGAGCTTGACTT	AAATCATGCCCATTAGCTGC
C/EBPβ	NM_131884.2	CTACCTCGCTTTCCACAGCA	TCGCTGTAGATTCCCAGTGC
SREBP1	NM_001105129.1	CAGAGGGTGGGCATGCTGGC	ATGTGACGGTGGTGCCGCTG
ChREBP	XM_001338467.2	ATCCCAGGGAGATCGCTCAT	TGCTGCTGGTTAAGGGAGTG
DGAT2	NM_001030196	TGGGGCTTTTTGTAACTTCG	TCTTCCTGGTGCACAGTCC
FASn	XM_682295	ATCTGTTCCTGTTCGATGGC	AGCATATCTCGGCTGACGTT
ACCα	NM_001271308.1	AGAGAGGGCAGGTTTTACCA	GCCATCATACGAGAGCAACA
ACOX1	BC097101.1	GCACGGATGTGTGTACCGTGC	GCGTCCAGAGCCCCTTGACCT
11β-HSD2	NM_212720.2	CCTTCAGGTGAGCATCCCTT	CCTCCAGAAGGTTTGGGGAC
11β-HSD3α	NM_200323.2	ATGCCATCGAACAGCTTGGA	AATTCACCTCTAGTAGCCATCG
IGF-I	NM_131825.2	GGCAAATCTCCACGATCTCTAC	CGGTTTCTCTTGTCTCTCTCAG
IGF-Iiα	NM_131433.1	TCGTGTGTGGAGAAGATGGC	ATGGTGTCCTTGTGAAGAGCC
β-actin	NM_181601.3	ACTGTATTGTCTGGTGGTAC	TACTCCTGCTTGCTAATCC
rpl8	NM_200713.1	TTGTTGGTGTTGTTGCTGGT	GGATGCTCAACAGGGTTCAT
