# Supplementary material for: The Mammalian “Obesogen” Tributyltin Targets Hepatic Triglyceride Accumulation and the Transcriptional Regulation of Lipid Metabolism in the Liver and Brain of Zebrafish
Source: PLoS One. 2015 Dec 3;10(12):e0143911. doi: 10.1371/journal.pone.0143911 (PMC4669123; doi:10.1371/journal.pone.0143911)
Supplement: S4 Table — (PDF) [file pone.0143911.s006.pdf]

S4 Table. Principal Component Analysis of the liver gene expression profile in TBT-exposed zebrafish.

**Gene correlations**	**PC1** (24.1%)	**PC2** (17.9%)	**PC3** (11.0%)	**PC4** (10.0%)	**PC5** (6.8%)
RXRα/a	0.610*	-0.447*	-0.062	0.380*	-0.017
PPARγ	0.589*	-0.029	0.308*	0.365*	-0.308*
DGAT2	0.673*	-0.426*	0.006	0.066	0.188
FASn	0.306	0.771*	0.159	0.142	0.022
C/EBPα	0.347	-0.051	0.384*	-0.659*	-0.172
C/EBPβ	0.567*	-0.422*	-0.289*	-0.064	0.300*
ACOX1	0.401*	0.212	0.331*	-0.340*	0.155
ACCα	0.462*	0.586*	-0.150	0.038	-0.055
SREBP1	-0.134	0.826*	0.146	0.293*	0.010
ChREBP	0.361	-0.071	0.446*	0.545*	0.267
IGF-I	0.637*	0.243	-0.447*	-0.163	-0.094
IGF-IIα	0.646*	-0.031	0.006	-0.083	-0.604*
11β-HSD2	0.150	0.282	-0.792*	0.128	0.003
11β-HSD3α	0.550*	0.367	0.052	-0.310*	0.485*
**TBT (ng/L)**			**Average scores on PCs**		
Males					
Control	0.293	0.0954	0.6121	0.1895	0.4146
10	0.8401	0.7524$^{\#}$	0.1329	0.4282	-0.7398$^{\#}$
50	-1.794$^{\#}$	2.0617	0.897$^{\#}$	0.1949	0.0504
Females					
Control	0.1094	-0.5101	-0.1473	-0.6059	0.3139
10	0.9133	-1.7933†	0.0927	0.4864	-0.4777
50	-0.3619	-0.4151	-0.3632	-0.3141	0.4386

*indicates significant correlations and $^{\#\dagger}$significant differences compared to the control (n=8, $^{\#}p<0.05$, $^{\dagger}p<0.1$; one-way ANOVA followed by Dunnett's test)
