# Supplementary material for: The Mammalian “Obesogen” Tributyltin Targets Hepatic Triglyceride Accumulation and the Transcriptional Regulation of Lipid Metabolism in the Liver and Brain of Zebrafish
Source: PLoS One. 2015 Dec 3;10(12):e0143911. doi: 10.1371/journal.pone.0143911 (PMC4669123; doi:10.1371/journal.pone.0143911)
Supplement: S5 Table — (PDF) [file pone.0143911.s007.pdf]

S5 Table. Principal Component Analysis of the brain gene expression profile in TBT-exposed zebrafish.

**Gene correlations**	**PC1** (28.1%)	**PC2** (15.2%)	**PC3** (10.4%)	**PC4** (9.5%)	**PC5** (7.3%)
RXRα/a	0.692*	0.0197	0.448*	-0.2111	0.1965
PPARγ	0.0557	0.5354*	0.1227	-0.5517*	0.2979
DGAT2	0.4546*	0.278	0.6361*	0.2442	-0.1452
FASn	0.8679*	-0.246	-0.0803	0.0073	0.1142
C/EBPα	0.7077*	0.3657	-0.1426	0.1436	0.1898
C/EBPβ	-0.1968	0.5737*	0.1577	-0.2567	0.3772*
ACOX1	0.6966*	-0.1349	0.0291	-0.2656	-0.0978
ACCα	0.8134*	0.0577	-0.1067	0.0905	-0.3071*
SREBP1	0.4417*	-0.3527	-0.4722*	0.2334	0.5201*
ChREBP	0.7317*	0.028	0.0749	0.0716	-0.131
IGF-I	-0.1795	-0.2139	0.4257*	0.6146*	0.4865*
IGF-IIα	-0.0861	0.5393*	0.0679	0.2879	-0.0206
11β-HSD2	-0.1781	-0.6243*	0.5882*	-0.0919	-0.0886
11β-HSD3α	-0.038	0.6041	-0.0768	0.4738*	-0.2139
**TBT (ng/L)**			**Average scores on PCs**		
Males					
Control	1.2452	1.0309	0.5326	0.2819	-0.3656
10	-1.3753#	-0.2094	-0.5757	-0.5171	-0.4775
50	0.764	-0.9313#	-1.4772#	0.8872	0.4224
Females					
Control	1.159	0.7599	0.6676	0.1025	-0.3231
10	-1.9967#	0.1849	0.3335	-0.5143	0.3634
50	0.2037	-0.835#	0.5191	-0.2402	0.3804

*indicates significant correlations and #significant differences compared to the control (n=8, $p<0.05$; one-way ANOVA followed by Dunnett's test)
