# Supplementary material for: The Mammalian “Obesogen” Tributyltin Targets Hepatic Triglyceride Accumulation and the Transcriptional Regulation of Lipid Metabolism in the Liver and Brain of Zebrafish
Source: PLoS One. 2015 Dec 3;10(12):e0143911. doi: 10.1371/journal.pone.0143911 (PMC4669123; doi:10.1371/journal.pone.0143911)
Supplement: S6 Table — (PDF) [file pone.0143911.s008.pdf]

S6 Table. Hepatic organotin levels. Tributyltin (TBT), Dibutyltin (DBT) and Monobutyltin (MBT) levels in the liver of male and female zebrafish following chronic TBT exposure (9 months).

(µg/g w.w.)	MBT	DBT	TBT
Males			
Control (n=1)	n.d.	n.d.	n.d.
TBT 10 ng/L (n=1)	1.1	7.3	3.0
TBT 50 ng/L (n=2)	4.0	23.1	8.2
Females			
Control (n=1)	n.d	n.d.	n.d.
TBT 10 ng/L (n=1)	2.2	7.9	2.0
TBT 50 ng/L (n=3)	5.3	28.8	8.8

Spare fish liver tissues from each exposure condition were pooled together to reach 0.05 g of dried sample; values are expressed in wet weight. Detection limits of assay (ng/g d.w.): MBT (232); DBT (132); TBT (75)
