# Supplementary material for: The Mammalian “Obesogen” Tributyltin Targets Hepatic Triglyceride Accumulation and the Transcriptional Regulation of Lipid Metabolism in the Liver and Brain of Zebrafish
Source: PLoS One. 2015 Dec 3;10(12):e0143911. doi: 10.1371/journal.pone.0143911 (PMC4669123; doi:10.1371/journal.pone.0143911)
Supplement: S1 Fig — (PDF) [file pone.0143911.s009.pdf]

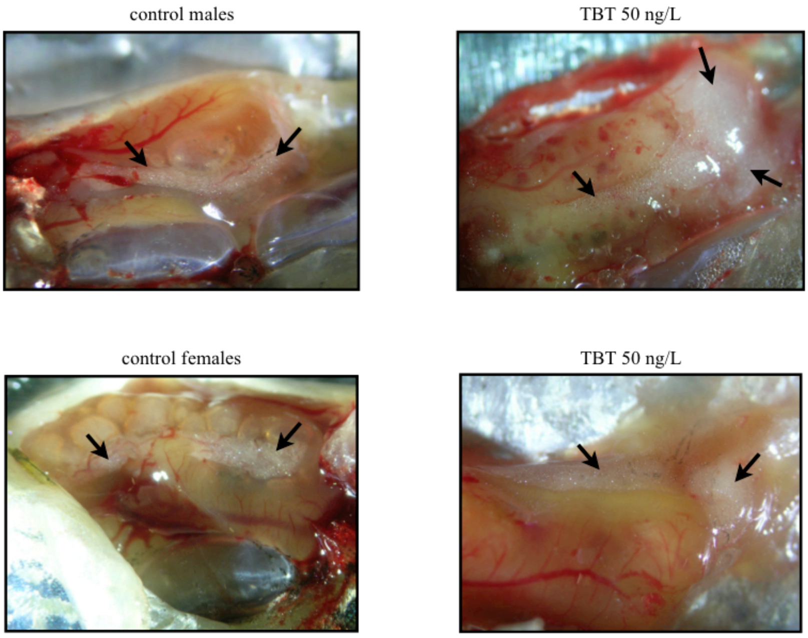


**S1 Figure. Visceral fat in TBT exposed zebrafish.** Zebrafish males and females exposed to TBT (50 ng/L as Sn) during their life cycle had visually more fat in the viscera than non-exposed fish.
